# Neglected Bicondylar Fracture of the Distal Humerus Treated With Total Elbow Arthroplasty

**DOI:** 10.7759/cureus.61818

**Published:** 2024-06-06

**Authors:** Platon Papageorgiou, Vasileios Giannatos, Antonios Batis, Zinon Kokkalis

**Affiliations:** 1 Orthopaedics and Traumatology, University of Patras, Patras, GRC; 2 Orthopaedic Surgery, Medical School, University of Patras, Patras, GRC

**Keywords:** non-union, elbow, tea, total elbow arthroplasty, distal humerus fracture

## Abstract

A 69-year-old woman suffered a distal humerus fracture including the medial and lateral condyles. She received conservative treatment with a posterior arm splint at a local healthcare center where she was evaluated by a non-specialist physician. Eight months later, she presented to our department complaining about severe instability. An upper limb specialist examined the patient at the time, and after thoroughly explaining the condition, he suggested a surgical approach with total elbow arthroplasty (TEA), which was then performed. At the one-year follow-up, the patient had a full range of motion without any complications or pain complaints. TEA in neglected fractures of the distal humerus is a poorly researched topic in the field of upper limb surgery with only scarce literature available. In this case report, we present the excellent outcomes of the procedure performed on an elderly patient after non-union regaining her quality of life and suggest that TEA can be a viable solution in elderly patients with complicated or non-united elbow fractures.

## Introduction

Distal humeral fractures constitute 2% of all adult fractures [1]. Most commonly, distal humerus fractures occur in elderly female patients, such as in our case, with underlying osteoporosis [2]. The first-line treatment is primarily surgical, consisting of open reduction and internal fixation (ORIF) [3]. Conservative treatment may be used in cases where the patient is unfit for an operation [4]. Additionally, missed or mistreated bicondylar fractures of the distal humerus are even rarer, which leads to a more challenging treatment and a lack of standard protocols for guidance [5]. In total elbow arthroplasty (TEA), two types are used: the semi-constrained, indicated for trauma cases where bone stock is limited, and unconstrained, indicated when sufficient bone stock and soft tissue exist for soft tissue balancing [6]. We present a case of neglected bicondylar fracture of the distal humerus which was treated with TEA, after being diagnosed with non-union of the fractured humerus. The objective was for the patient to gain back a full range of motion (ROM) without major restrictions. To do so, we had to keep intact the medial and lateral columns that create the triangle of the distal humerus, as well as the healthy structures of the elbow area that play a vital role in the functionality of the joint. Due to the lack of evidence on TEA for non-united elbow fractures, we present a very successful case treated with TEA after a non-united distal humerus fracture.

## Case presentation

**Figure 1:**
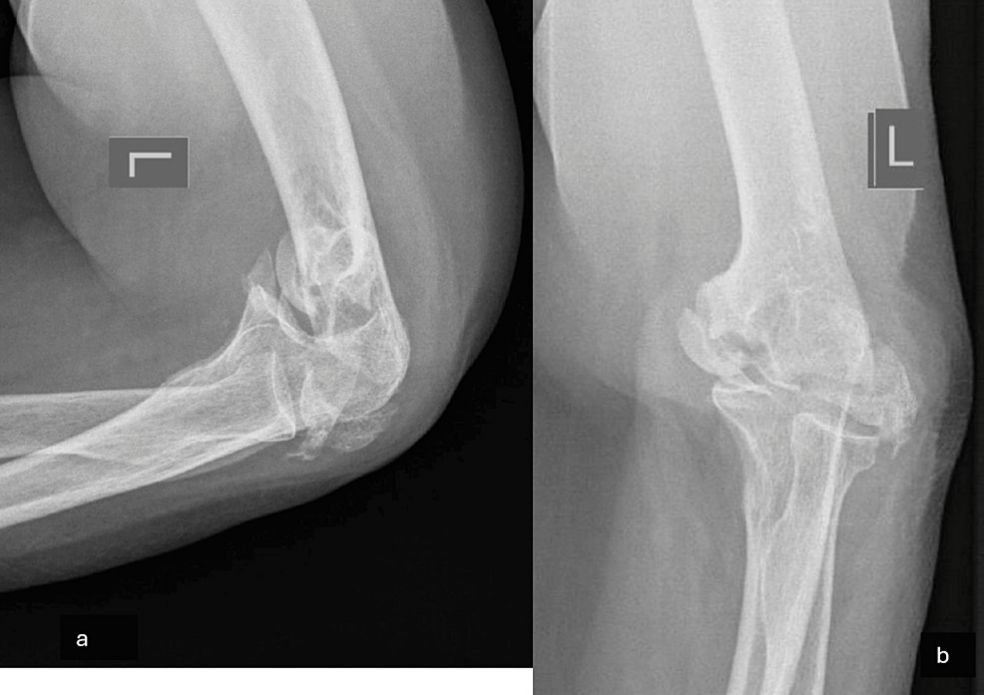
(a) Lateral elbow X-ray demonstrating the condyles. (b) Anteroposterior distal humerus X-ray demonstrating the non-union of the condyles.

**Figure 2:**
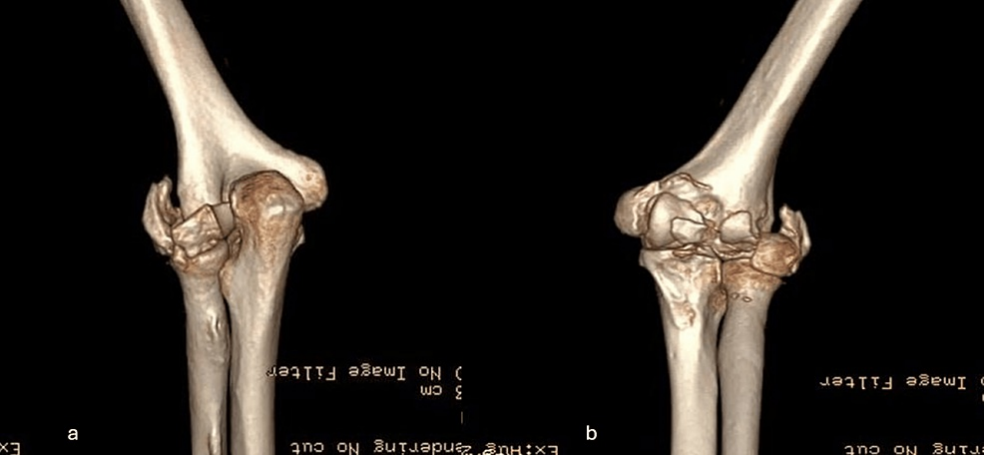
Three-dimensional CT scan of the elbow before the procedure showing the non-union of the lateral condyle and the valgus deformity of the elbow: (a) posterior view and (b) anterior view of the joint.

A 69-year-old woman presented at our hospital complaining of major instability and incapability of performing everyday tasks, accompanied by pain during activities but with a good ROM on her left elbow for the past six months. She recalled a history of a fall approximately eight months ago when she went to the local hospital and was diagnosed with a distal humerus bicondylar fracture. The fracture was treated conservatively and she was discharged with a posterior elbow splint. After experiencing incapacitating pain during everyday tasks, anteroposterior and lateral elbow X-rays were performed. She was diagnosed with a non-union of the bilateral condyles (Figures 1a, 1b). A CT scan was obtained to fully visualize the intra-articular pathology and develop a surgical plan (Figures 2a, 2b). We noticed an extensive absorption of the humeral condyles and severe corrosion of the remaining bone. A neurovascular examination was unremarkable. The physical examination showed major instability of the joint, without signs of stiffness. After a discussion with the patient and thoroughly explaining the potential benefits and caveats, the choice of surgical management with a TEA approach was made.

**Figure 3:**
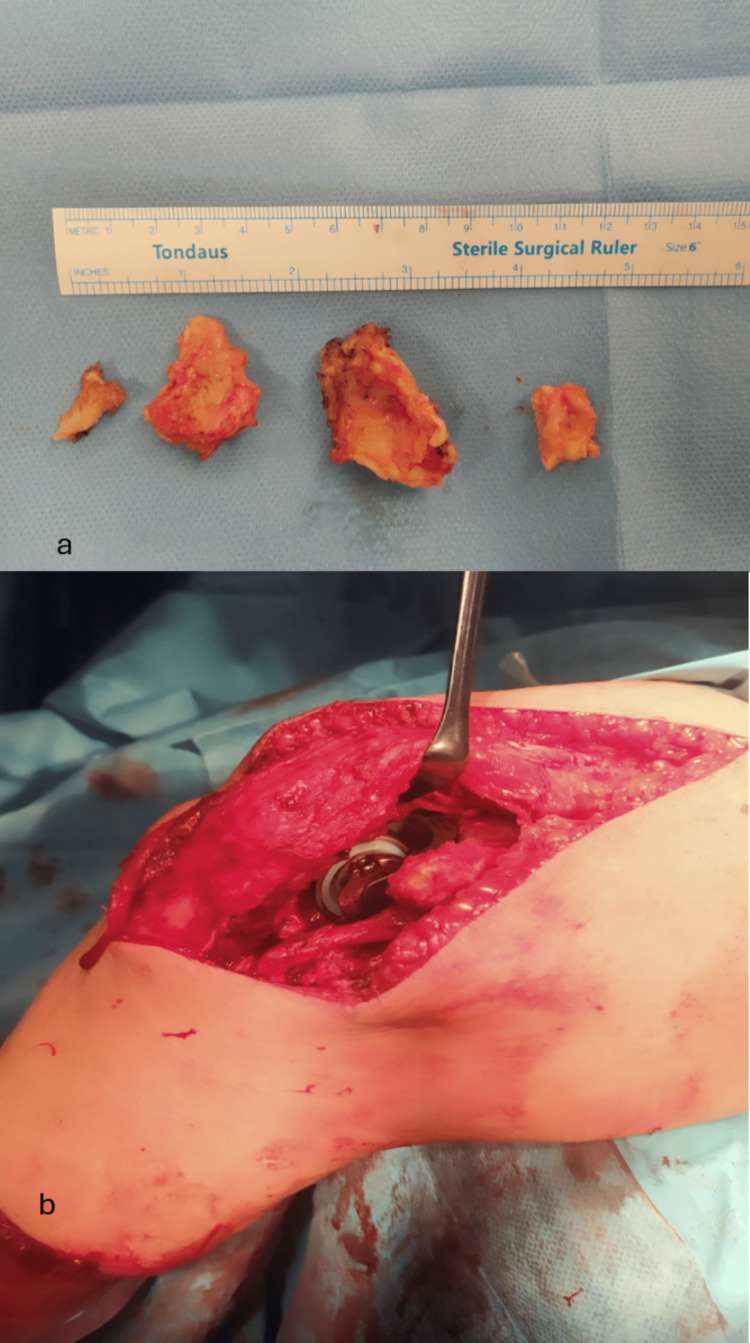
(a) Intraoperative image showing the osteochondral remnants of the medial and lateral epicondyles. (b) Intraoperative image showing the prostheses; note the triceps on approach.

The patient was placed in a decubitus (lateral) position on the right side with the left arm supported by a leg holder. The operation was performed under general anesthesia with the use of a tourniquet control at the left upper limb. A long posterior incision was carried laterally over the olecranon and we gained access to the articulation with the triceps on approach. The ulnar nerve was dissected and protected throughout the procedure. This approach released the lateral and collateral sides from the humerus with complete detachment of the flexor-pronator and ulnar collateral ligament origins from the medial epicondyle. Similarly, we detached the extensor-supinator and lateral collateral ligament origins on the lateral side. Following the first maneuver, we had full exposure on the radial head with detachment of the annular ligament. Afterward, we continued by removing the radial head and the tip of the olecranon using an oscillating saw. Then, the osteochondral remnants of the medial and lateral epicondyles were removed. We could clearly observe the corrosion that had occurred and worsened before the TEA (Figure 3a). The elbow joint was dislocated and both the distal humerus and proximal ulna were exposed for the TEA. An intraoperative fracture of the lateral condyle was stabilized with the insertion of a 3.5 mm cortical screw. Then, a typical semi-constrained total elbow arthroplasty was performed using Biomet Discovery Elbow System implants (Zimmer Biomet, St. Warsaw, Indiana USA). Both humeral and ulnar implants were fixated with cement (Figure 3b). After the implantation of the prosthesis, the elbow joint was very stable and had a full ROM. The ulnar nerve was left in place. Closing up the operation, layered stitching was performed, a drain tube was placed, and a simple bandage dressing was applied. The patient was discharged 48 hours after the procedure and was instructed to start active movement immediately.

**Figure 4:**
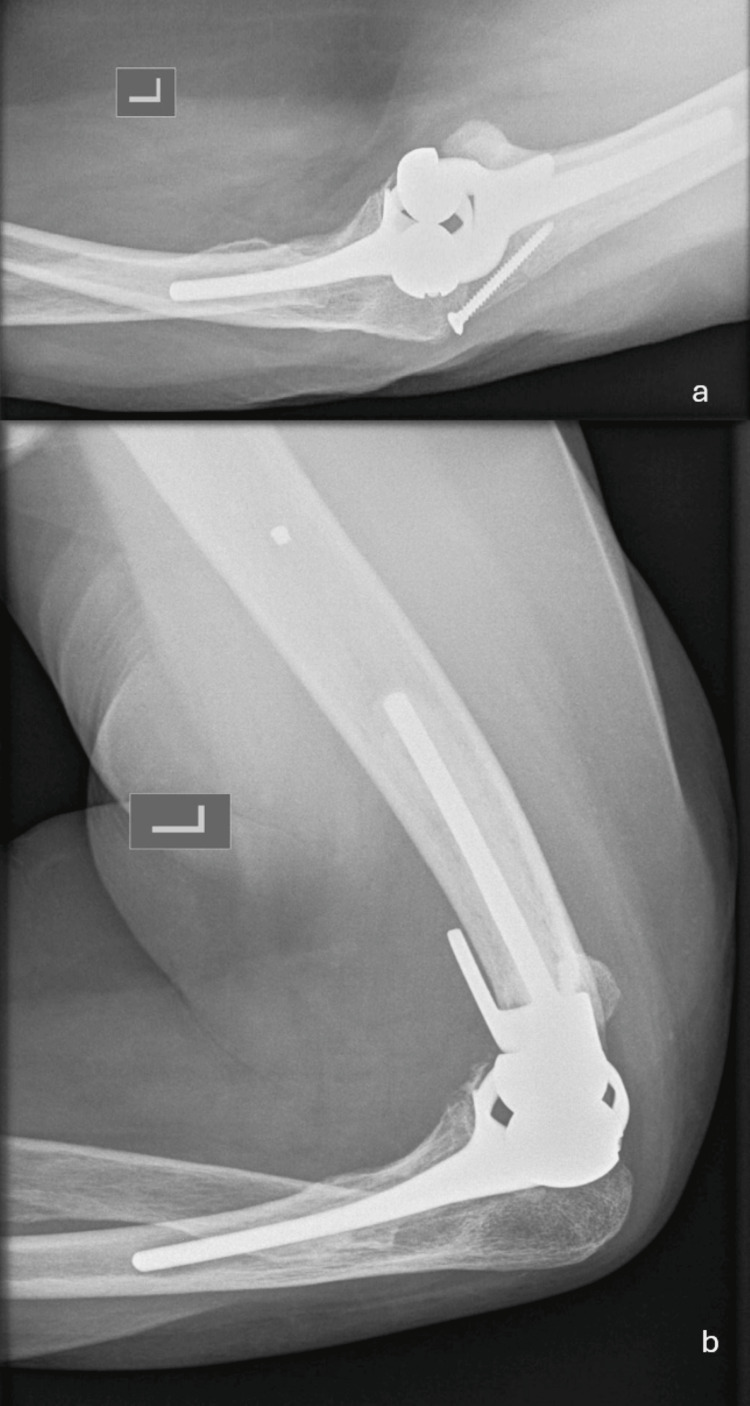
(a) Anteroposterior and (b) lateral X-rays at the six-month follow-up.

**Figure 5:**
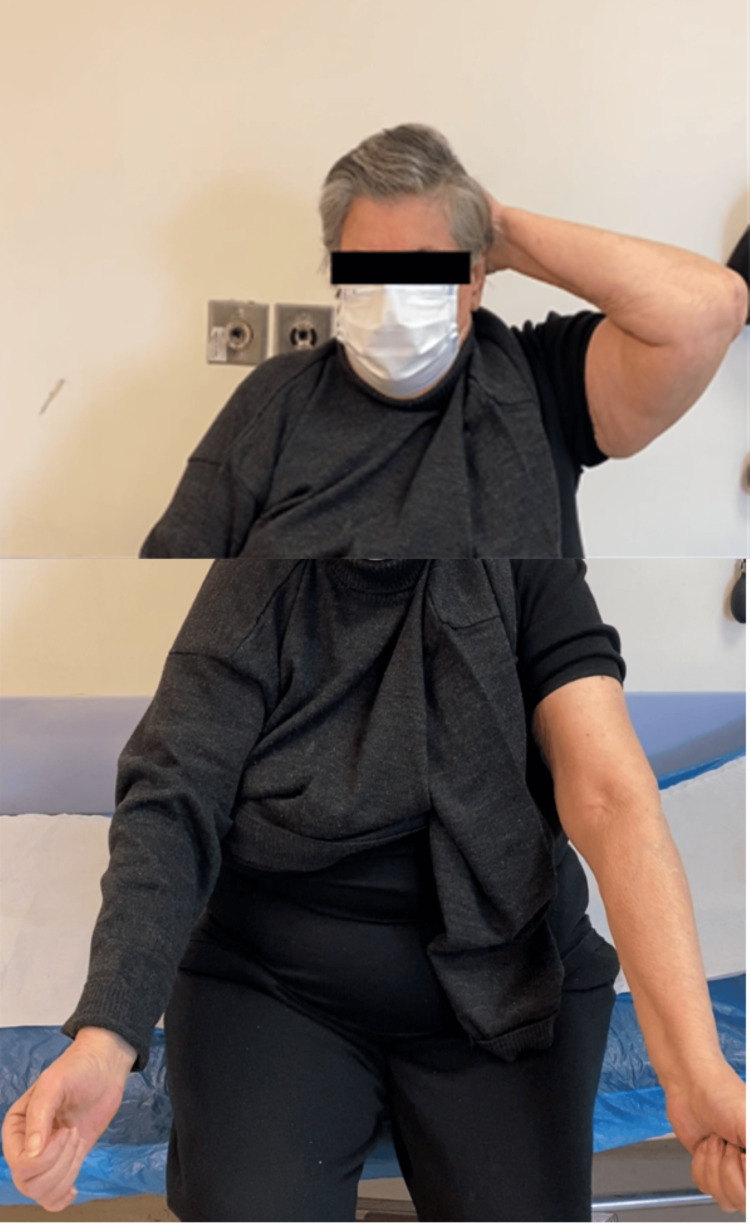
Clinical result six months after surgery with complete flexion and extension of the left elbow.

One month after the surgery, the joint had regained a full ROM. The X-rays obtained at the six-month follow-up are shown in Figure 4. At three, six, and twelve months of follow-up, the patient showed excellent results. She did not complain of any pain and returned successfully to her daily activities (Figure 5).

## Discussion

Bicondylar fractures of the humerus comprise a rare and challenging phenomenon in orthopedics [7]. Numerous classifications have been proposed through the years [5,8], with the most used ones being the Arbeitsgemeinschaft für Osteosynthesefragen/Orthopedic Trauma Association (AO/OTA) for general classification and the Jupiter-Mehne for bicondylar fractures. According to the AO/OTA classification, the case was classified as a Type C1 fracture. Our patient’s fracture was classified as Type low-T according to the Jupiter-Mehne classification. Low-T fractures are particularly difficult to manage because they typically consist of smaller fragments and can increase the risk of more severe postoperative complications such as avascular necrosis of the trochlea [9,10].

Taking into consideration the balance of risk between a TEA (including late infection and the need for revision of the procedure) and the benefits in comparison to the post-traumatic non-united elbow of the patient with major functional deficiency, we proceeded to preserve the integrity of the triceps. Preserving the triceps in continuity suggests a specific advance in the surgical technique as it allows immediate active joint movement without the fear of direct implant complications [2,8]. Both medial and lateral condyles and epicondyles had to be removed. However, condylar resection has a minimal clinically significant effect on the forearm, wrist, and hand strength and has no effect based on the Mayo Elbow Performance Score; it is ideal for certain conditions [11]. A great challenge in TEA is to keep the columns of the distal humerus intact. Therefore, a bur was needed to smooth the edges left by the saw to avoid a stress riser and prevent a columnar fracture [12].

Despite the exemplary surgical solution yet to be determined, TEA is considered a promising alternative and has been used more frequently, especially among the elderly [13,14]. Internal fixation is a procedure that demands extreme caution in these patients because of osteoporosis, poor-quality soft tissue, metaphyseal comminution, and intolerance to joint immobilization [15]. Nonetheless, even in cases with post-ORIF complications for distal humerus fracture, TEA seems to be a valuable alternative, particularly when addressing complications such as non-union, malunion, post-traumatic arthritis, and post-traumatic instability (mostly occurring in the elderly population), with the literature describing good results in 85% to 90% of patients at 5 to 10 years post-surgery, respectively [8,10]. Several studies suggest that primary TEA provides better results than ORIF in the elderly population based on functional outcome scores and ROM [16,17]. One of the most frequent complications is postoperative ulnar neuropathy which was a major concern in our case [16]. This complication is notably more often encountered in ORIF than in TEA [18]. In addition, a triceps-sparing approach is associated with less probability of ulnar neuropathy than a triceps-detaching approach [19]. For that reason, in our case, after we classified the fracture accordingly and based on the indications of the literature for distal humeral fractures in the elderly population, we selected TEA, especially the triceps on approach, as the treatment of choice which produced excellent functional results [1,20].

## Conclusions

Fractures of the distal humerus are challenging to treat. Moreover, in older patients who may have decreased sensitivity and lower functional demands, these fractures can remain undiagnosed for several months before the emergence of the symptoms. Total arthroplasty of the elbow remains a continually developing technique in modern medicine, with increasing indications in older patients. We conclude that, in the case of this patient with a non-united distal humerus fracture, the TEA through a triceps-sparing approach was a valuable alternative that allowed the patient to regain a fully functional elbow.

## References

[1] Beazley JC, Baraza N, Jordan R, Modi CS (2017). Distal humeral fractures-current concepts. Open Orthop J.

[2] Luchetti TJ, Abbott EE, Baratz ME (2020). Elbow fracture-dislocations: determining treatment strategies. Hand Clin.

[3] Rajaee SS, Lin CA, Moon CN (2016). Primary total elbow arthroplasty for distal humeral fractures in elderly patients: a nationwide analysis. J Shoulder Elbow Surg.

[4] Popovic D, King GJ (2012). Fragility fractures of the distal humerus: what is the optimal treatment?. J Bone Joint Surg Br.

[5] Bégué T (2014). Articular fractures of the distal humerus. Orthop Traumatol Surg Res.

[6] Edmonds A (2016). Elbow arthroplasty. Hand and Upper Extremity Rehabilitation (Fourth Edition).

[7] Court-Brown CM, Caesar B (2006). Epidemiology of adult fractures: a review. Injury.

[8] Ul Islam S, Glover AW, Waseem M (2017). Challenges and solutions in management of distal humerus fractures. Open Orthop J.

[9] Wiggers JK, Ring D (2011). Osteonecrosis after open reduction and internal fixation of a bicolumnar fracture of the distal humerus: a report of four cases. J Hand Surg Am.

[10] Morrey BF, Adams RA (1995). Semiconstrained elbow replacement for distal humeral nonunion. J Bone Joint Surg Br.

[11] Zalavras CG, Papasoulis E (2018). Intra-articular fractures of the distal humerus-a review of the current practice. Int Orthop.

[12] Helfet DL, Schmeling GJ (1993). Bicondylar intraarticular fractures of the distal humerus in adults. Clin Orthop Relat Res.

[13] Ducrot G, Ehlinger M, Adam P, Di Marco A, Clavert P, Bonnomet F (2013). Complex fractures of the distal humerus in the elderly: is primary total elbow arthroplasty a valid treatment alternative? A series of 20 cases. Orthop Traumatol Surg Res.

[14] Kokkalis ZT, Schmidt CC, Sotereanos DG (2009). Elbow arthritis: current concepts. J Hand Surg Am.

[15] Korner J, Lill H, Müller LP (2005). Distal humerus fractures in elderly patients: results after open reduction and internal fixation. Osteoporos Int.

[16] McKee MD, Veillette CJ, Hall JA (2009). A multicenter, prospective, randomized, controlled trial of open reduction--internal fixation versus total elbow arthroplasty for displaced intra-articular distal humeral fractures in elderly patients. J Shoulder Elbow Surg.

[17] Githens M, Yao J, Sox AH, Bishop J (2014). Open reduction and internal fixation versus total elbow arthroplasty for the treatment of geriatric distal humerus fractures: a systematic review and meta-analysis. J Orthop Trauma.

[18] Baik JS, Lee SH, Kang HT, Song TH, Kim JW (2020). Comparison of open reduction and internal fixation with total elbow arthroplasty for intra-articular distal humeral fractures in older age: a retrospective study. Clin Shoulder Elb.

[19] Dachs RP, Fleming MA, Chivers DA, Carrara HR, Du Plessis JP, Vrettos BC, Roche SJ (2015). Total elbow arthroplasty: outcomes after triceps-detaching and triceps-sparing approaches. J Shoulder Elbow Surg.

[20] Sanchez-Sotelo J (2011). Total elbow arthroplasty. Open Orthop J.

